# Treatment of Internal Hemorrhoids

**Published:** 1850-09

**Authors:** 


### SELECTIONS

FROM

### AMERICAN AND FOREIGN JOURNALS.

---

*Treatment of Internal Hemorrhoids.*—Dr. I. P. Garvin, in an interesting article in the Southern Medical and Surgical Journal (March, 1850,) states that he has treated a considerable number of cases of internal hemorrhoids, some of them very severe and of long standing, by the use of cold water, in the following manner:

He directs about a gill of cold water to be thrown into the rectum immediately before *every attempt* to evacuate the bowels, and that this enema be retained several minutes, if possible. This usually produces an evacuation of the feces, which have been so far softened on their surface, as to permit their escape without the least straining or irritation. After every evacution, it will be proper to use ablutions of the parts, more especially in such cases as are attended by some protrusion of the bowels.

This treatment is to be continued until some days after all uneasiness is removed. In old or very severe cases, to effect such amendments generally requires several weeks. It is highly important to impress upon the patient, the absolute necessity of perseverance in the use of cold water, even though he should be so far relieved as to feel *almost* well, for if it be suspended too soon, a very slight cause will bring on a relapse. So decided is the relief afforded by this treatment, that few persons would be disposed hastily to abandon it, but for the inconvenience of applying it daily. The ordinary apparatus for enemata are so unwieldy, that they cannot be carried about conveniently. All difficulty from this source may be obviated by the employment of a small pewter syringe with a ring handle to the piston. One which will hold two ounces is very convenient, and may be carried in the pocket, when necessary.

When such enemata of cold water fail to procure sufficient alvine evacuations, the quantity of fluid may be increased to half a pint, or it may be necessary to resort to mild laxatives. Active purgation must be carefully avoided. The patient should be advised, *never* to aid the natural expulsive action of the bowels by straining. If an evacuation cannot be procured without such efforts, it is best to postpone it until aided by the action of a laxative. If the convenience of the patient will permit, it will prove advantageous to change the usual hour for the daily defecation, to a regular hour in the evening—just before retiring for the night. This will obviate the gravitation of blood consequent upon the erect position.

This treatment will usually succeed equally well in hemorrhoids attended by hemorrhage. In this form of the disease, cold water will be found a most efficient astringent.

